# Implementation of earlier antibiotic administration in patients with severe sepsis and septic shock in Japan: antibiotic action needs time and tissue perfusion to reach target

**DOI:** 10.1186/s13054-020-2727-8

**Published:** 2020-01-14

**Authors:** Romain Jouffroy, Benoit Vivien

**Affiliations:** SAMU de Paris, Service d’Anesthésie Réanimation, Hôpital Universitaire Necker - Enfants Malades, Assistance Publique - Hôpitaux de Paris, and Université Paris Descartes - Paris 5, Paris, France

To the Editor:

We read with great interest the paper published in the Journal on November 19, 2019, by Abe et al. [1]. The authors reported not to retrieve any association between earlier antibiotic administration and reduction in in-hospital mortality of severe sepsis. First of all, the authors must be congratulated for their interesting work aiming to clarify the real impact of earlier antibiotic administration in septic shock, one of the key elements of care highlighted by the Surviving Sepsis Campaign (SSC) [2].

Nevertheless, to our opinion, some methodological issues deserve their results interpretation. From a statistical point of view, the categorization of the variable “time-to-antibiotic therapy” induces an information loss. Despite facilitating results interpretation, such categorization implies two consequences. First, it assumes that the treatment effect of antibiotic administration, from the 1st minute, if practically possible, to the 59th minute after diagnosis, is equivalent. Second, it would imply that the antibiotic therapy treatment effect is equivalent in all predefined categories, from 0–60 to 361–1440 min, which does not correspond to the reality, because the relationship between antibiotic therapy and mortality is not linear [3, 4]. In the present study, the negativity of the association between time to antibiotics (continuous variable) and mortality (OR = 0.999 [0.997–1.000]; *p* = 0.152) reflects this lack of linearity of the antibiotic therapy treatment effect. Furthermore, from a practical point of view, it is quite rare that the antibiotic therapy treatment effect is maximum since the first hour after administration.

Beyond this, to reach infected tissues, antibiotics need the restoration of a sufficient tissue perfusion pressure [5]. In their study, the authors [1] take into account the compliance rate to the first line of hemodynamic optimization (fluid expansion completed within 3 h) as a potential cofounder in their multivariate analysis but do not inform about the mean blood pressure (the reflect of tissue perfusion pressure) reached [2].

We fully agree with the authors that the impact of earlier antibiotic therapy is greater for most severe septic patients, but as reminded in the SSC, the outcome of these patients is not only dependent on a sole therapy but more from a bundle of care [2]. More than the completion of guideline principles, we believe that impact on outcome is strongly affected by achievement of objectives, especially when the gravity is higher. Among the objectives to be achieved, we think that early hemodynamic optimization and antibiotic administration are the two utmost treatments allowing to reduce septic shock mortality.

## Author’s response to letter “Implementation of earlier antibiotic administration in patients with severe sepsis and septic shock in Japan: antibiotic action needs time and tissue perfusion to reach target”

Toshikazu Abe

We appreciate the consideration and comments from the SAMU de Paris regarding our study.

Management of time data is one of the most important processes in “time to intervention” studies. We studied multiple different time intervals as we recorded time as a continuous variable; however, results with these values were not different from what we ultimately described. The relationship between time to antibiotic administration and mortality is not linear; therefore, we dealt with time data as a categorical variable. Hourly categorization is the most acceptable time interval used by clinicians. Because the number of patients receiving antibiotics after 361 min was small, we grouped those patients together. Our study did not mention causal inference, and it is a descriptive analysis using implementation science.

We did not show mean blood pressure, but we controlled tissue perfusion pressure by using the Sequential Organ Failure Assessment (SOFA), which includes a cardiovascular score. We also stratified patients by the presence or absence of shock. However, we did not find any association between time to antibiotic and outcomes with adjustment of those variables.

As you noted, the effect of antibiotics would be related to the time to administration and antibiotic sensitivity, concentration, and tissue perfusion. These variables may be even more important than time to administration. The lack of association between time to antibiotic administration and outcomes in our study may have been because of the lack of information about the variables. Other aspects of treatment may have differed among institutions, although we controlled for that using generalized estimating equations (GEE).

We believe that the effect of time to administration will be significant only when the overall quality of care is excellent. As with the differences for door to balloon time for acute coronary syndrome noted in the research by Menees and colleagues [6] and the research by Nallamothu and colleagues [7], the difference in quality may only be distinguished in highly standardized facilities.

A more accurate diagnosis may allow for better antibiotic choices, which is related to the outcome of time to antibiotic administration [8, 9]. Generally, antibiotics for meningitis should be administrated within 30 min, whereas antibiotics for infective endocarditis can wait for administration until culture results indicate the specific pathogen, as long as the patient’s vital signs are stable. Time recommendations for administration of antibiotics to patients with sepsis could be modified for different sites of infection as well as different clinical presentations, such as vague or apparent symptoms, and shock [10].

## Data Availability

Not applicable
